# The efficient generation of functional human hepatocytes from chemically induced pluripotent stem cells

**DOI:** 10.1111/cpr.13540

**Published:** 2023-10-09

**Authors:** Yun Lv, Ziyan Rao, Lulu Liu, Jun Jia, Chenyang Wu, Jun Xu, Yuanyuan Du, Yinan Liu, Bei Liu, Jihang Shi, Guangya Li, Dongyu Zhao, Hongkui Deng

**Affiliations:** ^1^ School of Basic Medical Sciences, MOE Engineering Research Center of Regenerative Medicine, State Key Laboratory of Natural and Biomimetic Drugs Peking University Health Science Center and the MOE Key Laboratory of Cell Proliferation and Differentiation, College of Life Sciences, Peking‐Tsinghua Center for Life Sciences, Peking University Beijing China; ^2^ Department of Biomedical Informatics, School of Basic Medical Sciences Peking University Health Science Center Beijing China; ^3^ State Key Laboratory of Vascular Homeostasis and Remodeling Peking University Beijing China; ^4^ Peking University‐Tsinghua University‐National Institute of Biological Science Joint Graduate Program, Academy for Advanced Interdisciplinary Studies, Peking University Beijing China; ^5^ Changping Laboratory Beijing China; ^6^ Department of Cell Biology, School of Basic Medical Sciences Peking University Stem Cell Research Center, Peking University Health Science Center, Peking University Beijing China; ^7^ Department of Hepatobiliary Surgery, First Medical Center of Chinese PLA General Hospital Beijing China; ^8^ Ministry of Education (MOE) Key Laboratory of Cell Proliferation and Differentiation, College of Life Sciences, Peking‐Tsinghua Center for Life Sciences Peking University Beijing China

## Abstract

Derivation of human hepatocytes from pluripotent stem cells in vitro has important applications including cell therapy and drug discovery. However, the differentiation of pluripotent stem cells into hepatocytes in vitro was not well recapitulated the development of liver. Here, we developed a differentiation protocol by mimicking the two‐stage development of hepatoblasts, which permits the efficient generation of hepatic progenitor cells from chemically induced pluripotent stem cells (hCiPSCs). Single‐cell RNA sequencing (scRNA‐seq) indicates the similarity between hepatoblasts differentiated in vitro and in vivo. Moreover, hCiPSC‐derived hepatic progenitor cells can further differentiate into hepatocytes that are similar to primary human hepatocytes with respect to gene expression and key hepatic functions. Our results demonstrate the feasibility of generating hepatic progenitor cells and hepatocytes from hCiPSCs with high efficiency and set the foundation for broad translational applications of hCiPSC‐derived hepatocytes.

## Graphical abstract

A new differentiation protocol mimicking the two‐stage development of hepatoblasts to potentiate their transition to hepatic progenitor cells (hiHPCs), which permits the high‐efficiency generation of hiHPCs from chemically induced pluripotent stem cells (hCiPSCs).

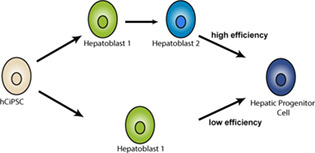

## 1 INTRODUCTION

The generation of induced human pluripotent stem cells (iPSCs) has shed light on the preparation of multiple types of functional cells by in vitro differentiation, indicating great application potential.^1^, ^2^, ^3^ Recently, our group successfully generated hCiPSCs from human somatic cells via small molecule‐based chemical reprogramming.^4^, ^5^ Chemical reprogramming is easily standardized and manufactured, non‐integrative to the genome, and cost‐effective, making hCiPSCs manufacturing appropriate for clinical application. Moreover, we have demonstrated the feasibility of differentiation of hCiPSCs into functional islets that ameliorate diabetes in non‐human primates^6^; however, there is little knowledge regarding the differentiation of this new stem cell type for the production of other functionally mature cells.

Hepatocytes have important applications in drug screening, disease modelling, and cell therapy for severe liver disease. In recent years, step‐wise protocols for the differentiation of pluripotent stem cells toward hepatocytes have been established by mimicking liver development,^7^, ^8^, ^9^, ^10^, ^11^, ^12^, ^13^ which can be generally divided into three stages: the generation of definitive endoderm, hepatoblast specification, and further differentiation toward mature hepatocytes.^14^ Previously, our group established a differentiation protocol to generate human induced pluripotent stem cells derived hepatic progenitor cells (hiHPCs) that can proliferate and further differentiate into human induced pluripotent stem cells derived hepatocytes (hiHEPs) with mature functions,^15^ allowing the preparation of large quantities of hepatocytes in vitro. Hepatoblasts are a crucial cell stage during the in vivo organogenesis of the liver^16^ and it has been reported that the development of hepatoblasts can be divided into two stages, after which successful differentiation into foetal hepatocytes occurs.^17^ However, there is only one stage of hepatoblasts in the existi differentiation protocols. Precisely mimicking the two‐stage development of hepatoblasts may potentiate the differentiation of pluripotent stem cells into hepatocytes in vitro.

In the present study, we mimicked the two‐stage development of hepatoblasts in vitro and established a differentiation protocol for the generation of hiHPCs from hCiPSCs. scRNA‐seq indicates the stage‐specific transcriptomic similarity between hCiPSC‐derived hepatoblasts and human foetal liver cells. In addition, these hiHPCs can further differentiate into hiHEPs possessing mature functions, which hold significant potential for clinical use.

## 2 MATERIALS AND METHODS

### 2.1 Cells and cell culture

Primary human hepatocytes (PHHs) were isolated from human donor hepatectomy. Firstly, PBE buffer solution (9 g/L NaCl, 0.42 g/L KCl, 2.1 g/L NaHCO3, 0.9 g/L glucose, 4.78 g/L HEPES and 0.37 g/L EDTA) was injected for about 1 hour. It was then replaced with PBCD buffer infusion (9 g/L NaCl, 0.42 g/L KCl, 2.1 g/L NaHCO3, 0.9 g/L glucose, 4.78 g/L HEPES, 0.25 g/L collagenase and 0.25 g/L dispase) until the liver tissue became no longer dense. The loose liver tissue was separated and hepatocytes were collected for the next experiment. HepG2 cell line was a gift from Kuanhui Xiang (Peking University Health Science Center) and were cultured in DMEM containing 10% FBS (Thermo Fisher Scientific).

### 2.2 hCiPSC culture and differentiation

Human adult hADSCs were isolated from adult adipose tissue that was obtained with informed written consent and approval by the Ethics Committee of Chinese PLA General Hospital (No. S2021‐557‐01) and the Institute of Ethics Committee Review Board in Peking University (IRB 00001052‐19070). The established human chemically induced pluripotent stem cell (hCiPSC) lines (C16, C19 & 10.7) were generated using chemical reprogramming protocol in our laboratory. These hCiPSC lines were cultured in mTeSR PLUS medium (Stem Cell Technology, 5825) on Matrigel (BD, 354277) coated plates and passaged with ReleSR (Stem Cell Technology, 05873) under 5% CO_2_ at 37°C. When the confluency of hCiPSCs reaches 70%~80%, the cells were replated using mTeSR PLUS medium containing 10 μM Y27632 (Selleck, S1049), 10ng/ml Activin A (Stemimmune, HST‐A‐0100), 10ng/ml bFGF (Peprotech, 100‐18B‐500) for one day. For inducing the differentiation of hCiPSCs into definitive endoderm, cells were treated with the combination of 100 ng/ml Activin A, 0.5 ng/ml BMP4 (Stemimmune, HST‐B4‐0100), 5 ng/ml bFGF, 10 μM Y27632, and 3 μM CHIR99021 (Selleck, S1263) for 1 day in RPMI1640 medium (Invitrogen, 22400‐089) with B27 supplement (GIBCO, 12587‐010), and then cultured in RPMI1640 medium with 100 ng/ml Activin A, 0.5 ng/ml BMP4, 10 μM Y27632, 5 ng/ml bFGF and B27 supplement (GIBCO, 12587‐010) for another 3 days. hCiPSCs derived definitive endoderm cells were further specified into foregut endoderm cells with the combination of 20 ng/ml KGF (Stemimmune, EST‐KF‐0100), 10 μM Y27632 and 2.5 μM SB431542 (Selleck, BCP01765) for 2 days in RPMI1640 medium with B27 supplement (GIBCO, 12587‐010). Then the foregut cells were differentiated into hepatoblasts 1 with the combination of 20 ng/ml KGF, 100 ng/ml BMP4, 20 ng/ml BMP2 (Stemimmune, 120‐02‐100), 10 μM Y27632 and 10 ng/ml bFGF for 3 days in RPMI1640 medium with B27 supplement (GIBCO, 12587‐010). At the end of this stage, the hepatoblasts 1 were replated and further maintained in the above medium supplemented with 10 μM Y27632 (Selleck, S1049) for 1 day. Then continue differentiation to hepatoblasts 2 was performed in William's medium E (Gibco, A1217601) with the combination of 25 μM forskolin (Topscience, T2939) and 20 ng/ml EGF (Peprotech, AF‐100‐15‐1000), 10 μM Y27632 and B27 supplement (GIBCO, 12587‐010) for 3 days. The hiHPCs would be generated and expanded in medium DMEM/F12 (Gibco, 11330‐032) mixed with William's medium E (Gibco, A1217601) in the ratio of 1:1 containing B27 supplement (GIBCO, 17504044) and 25 μM forskolin, 2.5 μM SB431542, 20 ng/ml EGF, 3 μM CHIR99021, 5 μM LPA (cayman chemical company, 62215), 1 μM dexamethasone (aladdin, D137736) and 0.5 μM S1P (Santa Cruz, sc‐201383C), 5mM Nicotinamide,100 μM PVC, 100μg/ml Heparin. Finally, hiHPCs were differentiated into mature hepatocytes with the Williams's medium E containing B27 supplement (GIBCO, 17504044), 50 μM forskolin and 10 μM SB431542.

### 2.3 Immunofluorescence

Cultured cells were washed with PBS and fixed in 4% paraformaldehyde at room temperature for 15 min. They were then washed 3 times with PBS for 3 min and then blocked with PBS containing 0.25% Triton X‐100 and 5% normal donkey serum (Jackson Immune Research Laboratories, Inc) at room temperature for 1 h or at 4°C overnight. The samples were incubated with primary antibodies at 4°C overnight, washed three times with PBS, and then incubated with the appropriate secondary antibodies for 1 h at room temperature in the dark. Nuclei were stained with DAPI (Roche). The primary and secondary antibodies used for immunostaining are listed in Supplementary information, Table S1.

### 2.4 mRNA expression analysis

Total RNA was isolated using the Direct‐zol RNA Miniprep Kit (ZYMO Research) and reverse‐transcribed with TransScript First‐Strand cDNA Synthesis SuperMix (TransGen Biotech). RT‐qPCR was performed using KAPA SYBR® FAST Universal qPCR Mix (KAPA Biosystems) on the BIO‐RAD CFX384TM Real‐time System. Quantified values were normalized to the input determined by house‐keeping genes (RRN18S). The RT‐qPCR primer sequences are provided in Supplementary information, Table S2.

### 2.5 Flow cytometry

Cells were dispersed into single‐cell suspensions with accutase and fixed in fixation (BD, 554714) at 4°C for 20 min and then washed 3 times with wash buffer (BD, 554723). Then the cells were incubated with primary antibodies at 4°C for 2h. After washed 3 times, the cells were incubated with secondary antibodies at at 4°C for 1h. Finally, the cells were washed 3 times and analyzed using a flow cytometer (Beckman CytoFlex).

### 2.6 Human albumin and urea detection

Human albumin was measured using the Human Albumin ELISA Quantitation kit (Bethyl Laboratory, E80‐129) according to the manufacturer's instructions. Urea synthesis was measured using the Urea Assay Kit (ABNOVA, KA1652) according to the manufacturer's instructions.

### 2.7 PAS staining, LDA uptake and red O staining

The PAS staining system was purchased from Sigma‐Aldrich. Cultures were fixed with 4% paraformaldehyde (DingGuo) and stained according to the manufacturer's instructions. For the LDL uptake assay, Heps were incubated with 10 μg/mL DiI‐Ac‐LDL (Invitrogen) for 4 h and 1 μg/mL Hoechst 33342 (Thermo Fisher Scientific) for 30 min at 37°C and then washed 3 times before imaging using fluorescence microscopy. Lipid detection was performed with a Lipid (Oil Red O) Staining Kit (Sigma) according to the manufacturer's instructions.

### 2.8 Measurement of CYP3A4 activity

The measurement of the CYP3A4 activity was performed with the P450‐Glo^TM^ CYP3A4 Assay kit (Promega, V9002) according to the manufacturer's instructions. Briefly, hepatocytes were washed with PBS for one time and incubated with the culture medium containing the substrate (Luciferin‐IPA) of CYP3A4 in 37°C for 1 hour. 25 μL medium was transferred into white 96‐well plates and mixed with 25 μL Luciferin Detection Reagent. After incubation in room temperature for 20 minutes, the luminescence was measured by SpectraMax® i3, the integration time was set to 1000 ms.

### 2.9 bulk RNA‐seq analysis

RNA‐seq raw reads were mapped to the human genome hg38 using HISAT2^1^ (v.2.2.0) with default parameters. SAM/BAM file formats were transformed by samtools^2^ (v.1.6). Expression value (number of raw reads) for each gene was determined by the software HTSeq^3^ version v.2.0.2 under the parameters ‐‐stranded=no and ‐‐mode= intersection‐nonempty. scRNA‐seq samples were treated as pseudo bulk RNA‐seq by summing UMI counts across all cells. We remained genes the sum of whose counts were more than 1 in at least 1 sample and normalized (the TMM method) expression values were determined by edgeR version 3.38.4 ran with an R version 4.2.0. Bulk RNA‐seq and scRNA‐seq samples were integrated using ComBat_seq function of the R package sva (v. 3.44.0) to remove batch effect. Samples of hCiPSC, hepatoblast, hiHPC, and hiHEP were used to calculate the stage highly expressed genes (also called stage‐specific genes) during the differentiation process. Stage‐specific genes were calculated using Wilcoxon rank‐sum (two‐sided) and p‐value adjustment was performed using the Benjamini–Hochberg correction (adjusted P < 0.05, logFC > 2). Functional hepatic lineage‐related gene lists were from a recent publication^4^. We visualized the expression of stage‐specific genes and functional genes by using R package pheatmap (v.1.0.12).

### 2.10 scRNA‐seq analysis

The reads of samples were aligned to the GRCh38 human genome provided by 10x Genomics (refdata‐gex‐GRCh38‐2020‐A) and pre‐processed using the Cell Ranger 10x Genomics software cellranger‐7.0.1. In this version, intronic reads are counted for whole transcriptome gene expression data. The filtered feature‐barcode matrices generated by Cell Ranger were used for downstream analyses. For quality control, we filtered out cells expressing fewer than 200 genes, fewer than 2,000 counts, more than 11,000 counts, and more than 10% counts in mitochondrial genes and genes expressed in fewer than 3 cells using SCANPY^5^ (v.1.9.1) in a Python (v.3.9.13) environment. Doublets were estimated by scrublet^6^ (v.0.2.3) with default parameters in scanpy. external. pp. scrublet function and removed.

### 2.11 Clustering of cells and differential gene expression analysis

We did downstream analyses in SCANPY (v.1.9.1). We normalized the data matrix to 10,000 reads per cell, making counts comparable among cells, and then did log1p transformation. Highly variable genes (HVGs) were selected by the thresholding of minimum mean expression 0.0125, maximum mean expression 3, and minimum dispersion 0.5. Then, we regressed out the effects of total counts per cell and the percentage of mitochondrial genes expressed and scaled the data to unit variance. Principal component analysis (PCA) was performed to get dimensionality reduction data and 30 PCs were used to create a neighborhood graph for the cells under the parameter neighbors=80. We used Leiden^7^ clustering to cluster cells with a resolution of 0.05 to distinguish between a main and small group. For combined data, we used harmonypy^8^ (v.0.0.6) to remove the batch effect and calculate a neighborhood graph for analysis. Uniform Manifold Approximation and Projection (UMAP)^9^ was used for visualization. Differentially expressed genes were calculated using Wilcoxon rank‐sum (two‐sided) and p‐value adjustment was performed using the Benjamini–Hochberg correction. The Database for Annotation, Visualization, and Integrated Discovery (DAVID, v2022q4)^10,11^ was used to do gene enrichment analyses about Kyoto Encyclopedia of Genes and Genomes (KEGG) and Gene Ontology Biological Process (GOBP).

### 2.12 Trajectory and hepatic developmental driver genes inference

We selected major continuous cell clusters and used scTour^12^ (v.0.1.3) to infer in‐vitro hepatic cell developmental pseudo time. We trained model 200 epoch with parameters alpha_recon_lode=1.0, alpha_recon_lec=0.0, and batch_norm=True, and got the latent space with parameters alpha_z=0.2 and alpha_predz=0.8 while larger alpha_predz is more representative of the extrinsic pseudo time ordering. 30 neighbors and the inferred latent space's first 5 dimensions were used to calculate a neighborhood graph, and UMAP was used for visualization of pseudo time, vector field generated from scTour's latent space, and gene expression. The driver genes were obtained according to the Pearson correlation between normalized gene expression and pseudo time, and we considered top genes as the most potential hepatic developmental driver genes.

### 2.13 Comparison between in‐vitro and in‐vivo scRNA‐seq

PCW samples came from B Wesley, B. T. et al.^13^ According to the annotation of this article, we only used cells annotated as liver hepatocytes and got processed data including 8332 cells from https://collections.cellatlas.io/liver-development. CS samples were collected from Xu, Y. et al.^14^, and we processed this data further in SCANPY by filtering cells expressing more than 5000 genes, and selected cells annotated as “hepatocyte” from GSE157329 (processed data), getting 4183 cells. We then combined our in‐vitro data and 2 in‐vivo data and also calculated HVGs with default parameters. Harmony PCA was used to remove the batch effect. When comparing in‐vitro and in‐vivo data, we implemented ClusterMap^15^ algorithm to get the matched in‐vivo stages of in‐vitro samples. We got marker genes in each dataset using Wilcoxon rank‐sum (two‐sided) and selected different top number genes ranked by z‐score as marker genes to input in ClusterMap. ClusterMap compares subclusters from different conditions via hierarchical clustering based on the binary expression patterns of marker genes. The purity tree algorithm is used to get matched subclusters by default cutoff 0.1. Similarity scores are determined by cluster results.

### 2.14 Identify hepatic‐related regulons

We used pySCENIC^16^ (v.0.12.0) in a Python (v.3.9.13) environment to analyze regulons in each matched in‐vivo and in‐vitro samples. We got transcription factors (TFs) from https://raw.githubusercontent.com/aertslab/pySCENIC/master/resources/hs_hgnc_tfs.txt to calculate the gene regulatory network by running the GRNBoost2 network inference algorithm. Motif to TF annotation was from https://resources.aertslab.org/cistarget/motif2tf/motifs‐v9‐nr.hgnc‐m0.001‐o0.0.tbl and genome ranking information was from https://resources.aertslab.org/cistarget/databases/homo_sapiens/hg38/refseq_r80/mc9nr/gene_based/hg38__refseq‐r80__10kb_up_and_down_tss.mc9nr.genes_vs_motifs.rankings.feather. We input these 2 files into next step to do motif enrichment, which relies on a ranking and recovery approach to retain significantly enriched modules, and then combine modules sharing the same TF into a single regulon. Finally, we performed AUCell to get each regulon score in each cell. The TF of a regulon overlapped with hepatic lineage‐related genes was regarded as the hepatic‐related regulon. Cytoscape (v.3.8.2, Java 11.0.6) was used to visualize the hepatic development regulatory network. DAVID (v2022q4) was used to do gene enrichment about GOBP.

### 2.15 Statistical analysis

For all measurements, ‘n’ represents the number of biological replicates. Unless described otherwise, standard statistical analyses were performed with Graph Pad Prism 7 using default parameters. All of the error bars represent SD.

## 3 RESULTS

### 3.1 Differentiation of hCiPSCs into hiHPCs through two‐stage induction of hepatoblasts

**FIGURE 1:**
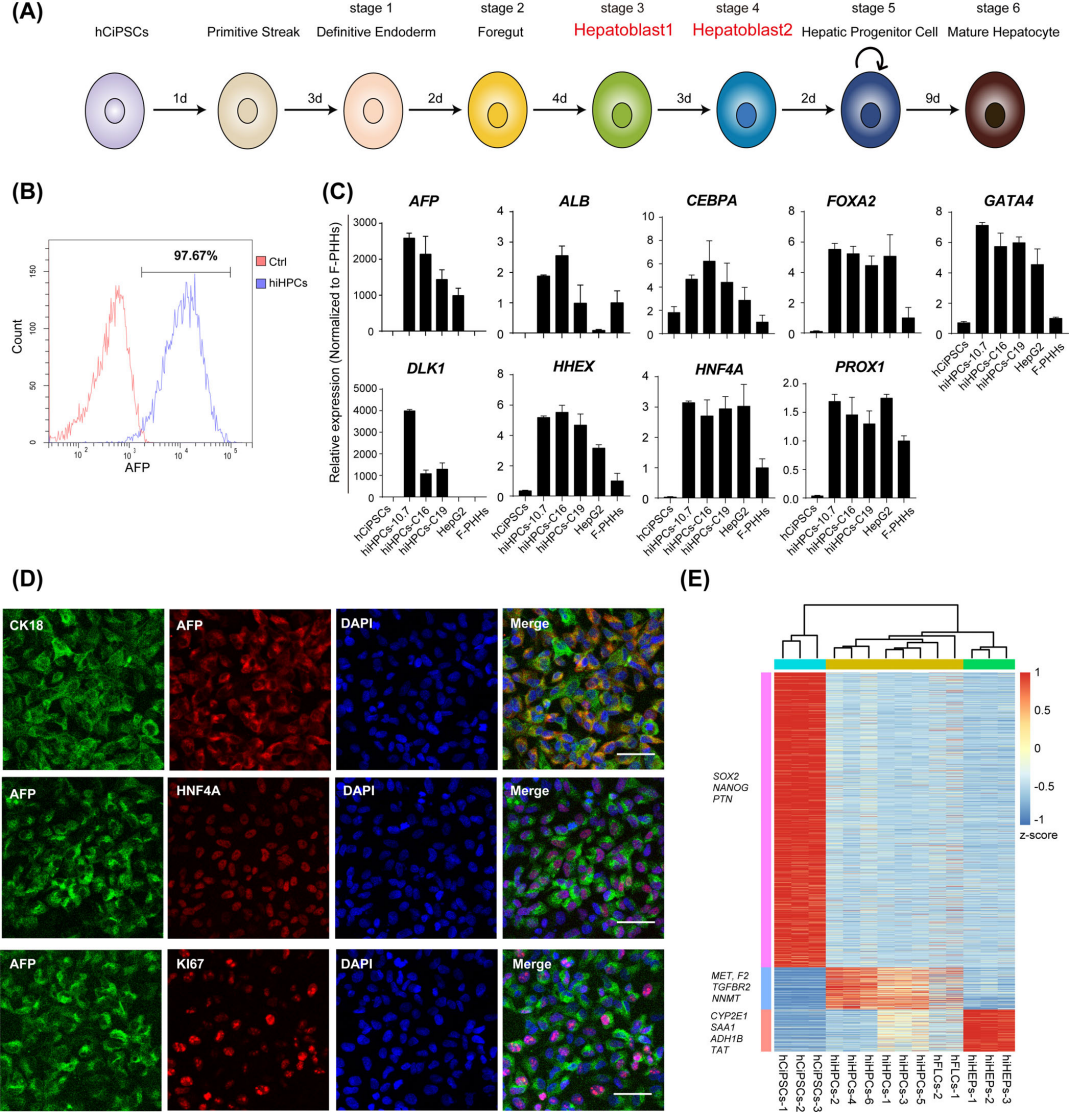
Differentiation and characterization of hCiPSC‐derived hiHPCs. (A) Schematic representation of the differentiation from hCiPSCs to hiHEPs. (B) Flow cytometry analysis of AFP‐positive hCiPSC‐derived hiHPCs. (C) RT‐qPCR analysis of the major hepatic progenitor genes in hCiPSCs (*n* = 3), hiHPCs (*n* = 3) derived from different hCiPSC lines, HepG2 cells (*n* = 3), and F‐PHHs (*n* = 3). Relative expression was normalized to F‐PHHs. (D) Co‐immunofluorescence staining of CK18, HNF4A, or Ki67 and AFP in hCiPSC‐derived hiHPCs. Scale bars: 50 μm. (E) Heatmap to show the expression patterns of stage‐specific genes in hCiPSCs, hiHPCs, hFLCs, and hiHEPs.

To better recapitulate the generation of hepatocytes from pluripotent stem cells, we focused on mimicking the two‐stage in vivo development of hepatoblasts to precisely regulate the cell fate of hepatoblasts prior to the generation of hiHPCs. Thus, based on our previously reported protocol,^15^ another differentiation stage of hepatoblast was added prior to the generation of hiHPCs (Figure 1A). During the early stage of expansion, the percentage of cells expressing α‐fetoprotein (AFP) was over 97%, suggesting a high purity of hiHPCs (Figure 1B). Additionally, the expression levels of multiple representative hepatic progenitor markers, including *AFP*, *ALB*, *FOXA2*, *GATA4*, *DLK1*, *HHEX*, and *HNF4A*, were confirmed in hiHPCs (Figure 1C). Further, immunofluorescence analysis revealed the co‐expression of AFP and CK18, HNF4A, and KI67 in hiHPCs (Figure 1D), verifying their hepatic progenitor properties at the protein level. Consistent with these observations, the heatmap showed that the stage‐specific gene expression profile of hiHPCs was indistinguishable from that of human foetal liver cells (hFLCs) and distinct from that of hCiPSCs and hiHEPs (Figure 1E). Besides, this differentiation protocol was also applicable to iPSCs induced by Yamanaka factors (Figure S1). Collectively, these results indicate that after the two‐stage differentiation of hepatoblasts, the hCiPSC‐derived hiHPCs possess high purity and key hepatic identity.

### 3.2 The two‐stage differentiation enhanced the hepatic signature of hepatoblasts

**FIGURE 2:**
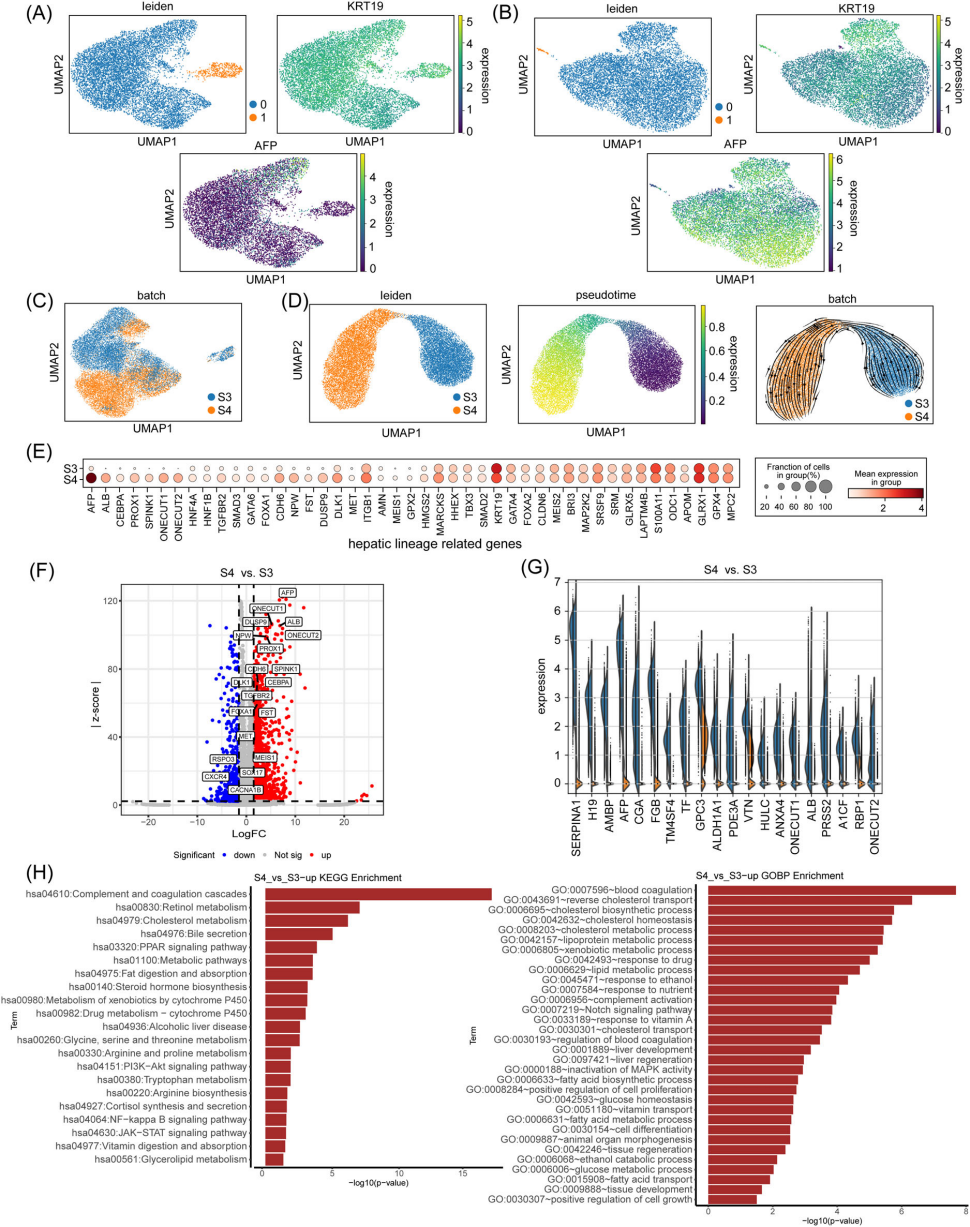
The gradual specification of hepatoblasts from Stage 3 to stage 4. (A) UMAP plots of the annotation of S3 cell population Leiden clusters (left) and the expression of hepatoblast markers KRT19 (right) and AFP (down). The expression of KRT19 and AFP was log‐normalized. (B) UMAP plots of the annotation of S4 cell population Leiden clusters (left) and the expression of hepatoblast markers KRT19 (right) and AFP (down). The expression of KRT19 and AFP was log‐normalized. (C) UMAP plot of combined S3 and S4 cells, coloured according to different stages. (D) UMAP plots of sctour trajectory inference of combined S3 and S4 cells (left), annotation of the estimated pseudotime (middle), and vector field (right). (E) Dot plot of the differential expression of hepatic lineage‐related genes between S3 and S4. The size of the circle represents the percentage of expressed cells. The colour shade indicates the mean expression of the gene at each stage. (F) Volcano plot of the overall differentially expressed genes between S3 and S4. Red dots represent the 1788 upregulated genes, while blue dots represent the 516 downregulated genes at S4 as compared with S3. The identified genes were hepatic lineage‐related genes. |logFC| > 1.5, Wilcoxon rank‐sum test adjusted *p*‐value <0.05. (G) Violin plot of the top 20 upregulated genes at S4 as compared with S3, ranked by *z*‐score. (H) KEGG (left) and gene ontology biological process (right) enrichment bar plot of the 1788 upregulated genes at S4 as compared with S3. The *x*‐axis represents the −log10 *p*‐value.

We utilized scRNA‐seq to examine the transcriptional characteristics of hCiPSCs‐derived hepatoblasts at Stage 3 and 4 to determine whether the hepatic signatures of hepatoblasts were enhanced after the additional stage. Hepatoblasts in Stage 3 and 4 exhibited a predominance in the expression of the hepatoblast marker KRT19, according to UMAP^18^ dimension reduction and Leiden^19^ clustering (Figure 2A,B). Besides, the expression of *AFP* was significantly upregulated in hepatoblasts at stage 4 (Figure 2A,B), suggesting potential enhancement of hepatic signatures of hepatoblasts at stage 4. Therefore, we looked more closely at the transcriptomic characteristics about cells at Stage 3 and 4. Combinatorial study revealed that a portion of differentiated cells at Stage 3 shared transcriptomic features with that at stage 4 whereas other cell populations showed stage‐specific transcriptomic features (Figure 2C). Then, we further performed pseudotime analysis scTour^20^ on the major cell groups to explore the gradual change of transcriptome of hepatoblasts at Stage 3 to that at stage 4 (Figure 2D). During the two‐stage differentiation, the expression levels of representative hepatic lineage regulators were gradually upregulated (Figure S2b), with several being driver genes responsible for the enhanced hepatic signature of hepatoblasts from Stage 3 to stage 4 (Figure S3).

To figure out the transcriptional differences between Stage 3 and stage 4 hepatoblasts, we first checked some hepatic lineage markers, and we observed that cells at stage 4 significantly upregulated multiple key hepatic regulators while maintaining hepatic lineage markers that were already highly expressed in cells at Stage 3 (Figure 2E). Next, we did differential gene expression analysis, and identified 1788 upregulated genes and 516 downregulated genes in stage 4 cells compared with Stage 3 cells (Figure 2F). The upregulated genes at stage 4 included several key hepatic lineage regulators such as ONECUT1, ONECUT2, PROX1, and CEBPA and several hepatic related genes were found among the top 20 upregulated genes (Figure 2G). KEGG and GO^21^ analyses showed that the upregulated genes at stage 4 were mainly involved in hepatic metabolic processes (Figure 2H), whereas the downregulated genes at stage 4 were primarily related to cell fate regulation of other lineages (Figure S2a). Collectively, these data suggest that the adjustment of the two‐stage differentiation enhanced the generation of hepatoblasts.

### 3.3 Transcriptomic similarities between hCiPSC‐derived hepatoblasts and foetal liver cells

**FIGURE 3:**
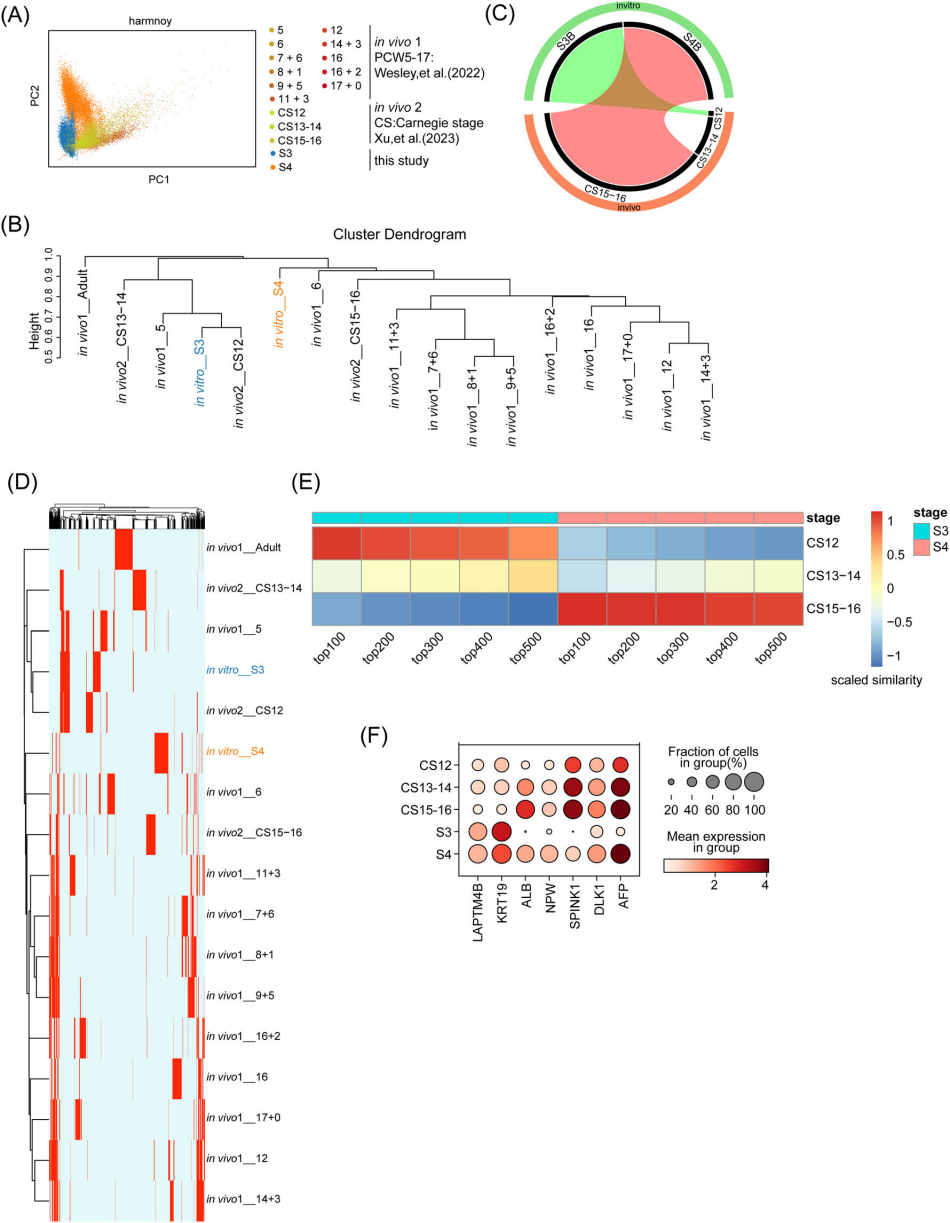
Hepatoblasts at Stage 3 and stage 4 can be matched to foetal liver cells at CS12 and CS15–16 in vivo, respectively. (A) Harmony PCA removal of the batch effect to show similarity among two in vivo and two in vitro scRNA‐seq datasets. CS, Carnegie stage; PCW, post‐conceptional week. (B) Hierarchical clustering of two in vivo and two in vitro scRNA‐seq datasets at different stages. (C) Circos plot of the matched groups between the two in vivo datasets and S3–4. The cord colour transparency indicates the scaled similarity of the matched groups. The percentage of cells in each sample is represented by the width of the black sectors. (D) Heatmap of marker expression in the two in vivo and two in vitro scRNA‐seq datasets at different stages. The red colour indicates the presence of a marker gene at that stage, and light blue indicates its absence. The hierarchical cluster of rows is the same as in (B). (E) Heatmap of the similarity between CS12–16 and S3–4 with respect to different numbers of marker genes. (F) Dot plot of hepatic lineage‐related genes whose direction of expression has the same tendency at both CS12–16 and S3–4. The size of the circle represents the percentage of expressed cells. The colour shade indicates the mean expression at each stage.

To estimate if two‐stage hepatoblasts differentiated in vitro were similar to human liver cells from early embryo, we compared the transcriptome of hCiPSCs‐derived hepatoblasts at Stage 3 and 4 to that of liver cells from embryo at post‐conceptional weeks (PCW) 4–17^17^ and Carnegie stages (CS) 12–16.^22^ CS 12 to 16 from the PC1 axis, which constituted 17.11% of highly variable genes, were shown to be similar to human liver cells in hCiPSC‐derived hepatoblasts according to principal component analysis (PCA) (Figure 3A). Further binary hierarchical clustering in ClusterMap^23^ was used to reveal that hCiPSCs‐derived hepatoblasts at Stage 3 resembled the liver cells from CS 12 or PCW5, whereas hepatoblasts at stage 4 were more similar to liver cells from CS 15–16 or PCW6 (Figure 3B). Considering the similarity of transcriptome cross whole development stages according to PC1 (Figure 3A), we only compared two‐stage hepatoblasts differentiated in vitro with CS 12–16, and came to the same conclusion supported by the ClusterMap own result (Figure 3C). These findings suggested that hCiPSCs‐derived hepatoblasts and human foetal liver cells have stage‐specific transcriptomic similarities.

As we know the similarity between hepatoblasts at Stage 3/4 and CS 12/15–16 and the difference between Stage 3 and 4 cells, we investigated if the transcriptomic differences between liver cells from CS 12 and CS 15–16 were also similar to that of Stage 3 and 4 cells. In total, we identified 92 up‐regulated and 30 down‐regulated genes in CS15‐16 compared with CS12 (Figure S4a,b). Up‐regulated genes were enriched in hepatocyte‐related pathways regarding cholesterol regulation, PPAR signalling, metabolism, and so on, while down‐regulated genes were enriched in other unrelated pathways in liver development (Figure S4c,d), consistent with the difference between Stage 3 and 4 cells. To assess the stability of similarity between Stage 3/4 cells and CS 12/15–16, we also utilized various marker gene counts. No matter how many marker genes there were, Stage 3 cells resembled CS12 the most, while stage 4 cells resemble CS15–16 (Figure 3D,E). Notably, the top differentially expressed genes included the hepatoblast markers *AFP* and *ALB* (Figure 3F). The expression of these marker genes showed a gradual increase in foetal liver cells from CS12 to CS15–16, a pattern similar to the upregulation of hCiPSC‐derived hepatoblasts from Stage 3 to 4. Collectively, these data suggest that foetal liver cells gradually acquire hepatoblast identity from CS12 to CS15–16, which was recapitulated during the in vitro differentiation of hCiPSCs to hepatic cells.

**FIGURE 4:**
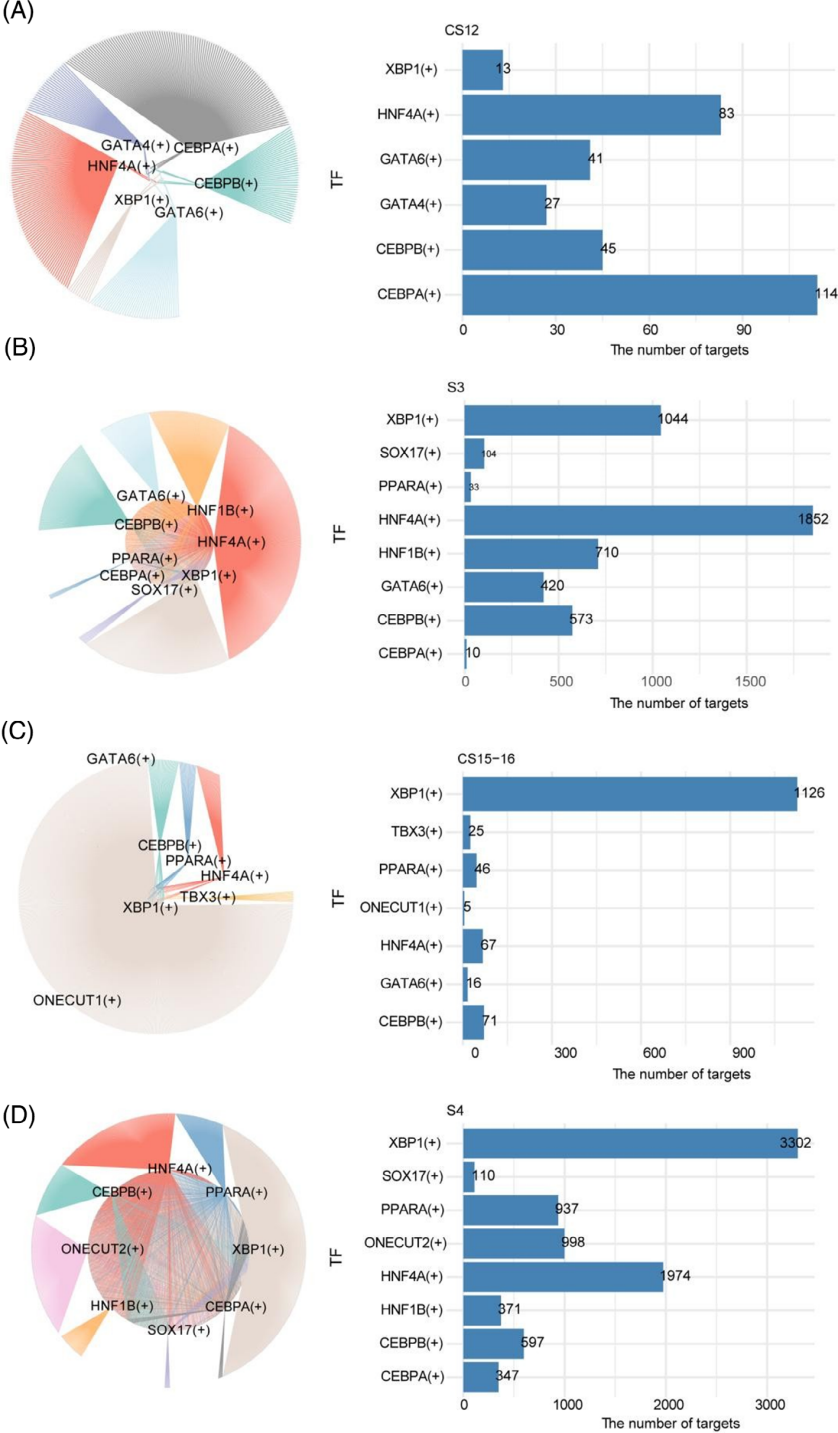
Analysis of hepatic regulon networks of hepatoblasts in vivo and in vitro. Hepatic‐related regulon network (left) coloured according to different regulons and the number of genes in each regulon (right) of hepatoblasts at CS12 (A), Stage 3 (B), CS15–16 (C), and stage 4 (D).

The transcriptomic differences in hCiPSC‐derived hepatoblasts between Stage 3 and 4 suggested potential differences in the core gene regulatory network between these two stages. We applied pySCENIC^24^ to examine the gene regulatory network of hepatoblasts at Stage 3 and 4 to confirm these differences, with foetal liver cells at CS12 and CS15–16 used as controls. Notably, we found that hepatoblasts at Stage 3 were enriched with regulons driven by XBP1, HNF4A, CEBPA, HNF1B, and GATA6, whereas those at stage 4 were enriched with regulons driven by XBP1, PPARA, ONECUT2, and HNF4A (Figure 4B,D). Control foetal liver cells at CS12 were enriched with HNF4A and CEBPA regulons, whereas those at CS15–16 were enriched with XBP1 regulons (Figure 4A,C). These results suggest that hCiPSC‐derived hepatoblasts and foetal liver cells share core regulons at the single‐cell level.

Finally, the biological processes regulated by the regulons in hCiPSC‐derived hepatoblasts at Stage 3 were evaluated (Figure S5). In hCiPSC‐derived hepatoblasts at Stage 3, HNF4A, CEBPB, and PPARA were involved in the regulation of hepatic metabolism, such as cholesterol synthesis and homeostasis, drug response, and glucose homeostasis, whereas SOX17, HNF1B, and GATA6 were involved in the regulation of developmental signalling pathways and morphogenesis (Figure S5). In addition, XBP1 was involved in the regulation of the cell cycle. Further, the biological processes regulated by the regulons in hCiPSC‐derived hepatoblasts at stage 4 were evaluated. Among these stage 4‐enriched regulons, XBP1, HNF4A, and PPARA were involved in the regulation of hepatocyte metabolic processes, whereas CEBPB and ONECUT2 were involved in hepatocyte differentiation and chromatin remodelling (Figure S6). Collectively, these results suggest that the hepatocyte program, which includes the pathways regulating hepatic metabolic function, is gradually activated in hCiPSC‐derived hepatoblasts.

### 3.4 Characterization of hiHPC‐derived hiHEPs

**FIGURE 5:**
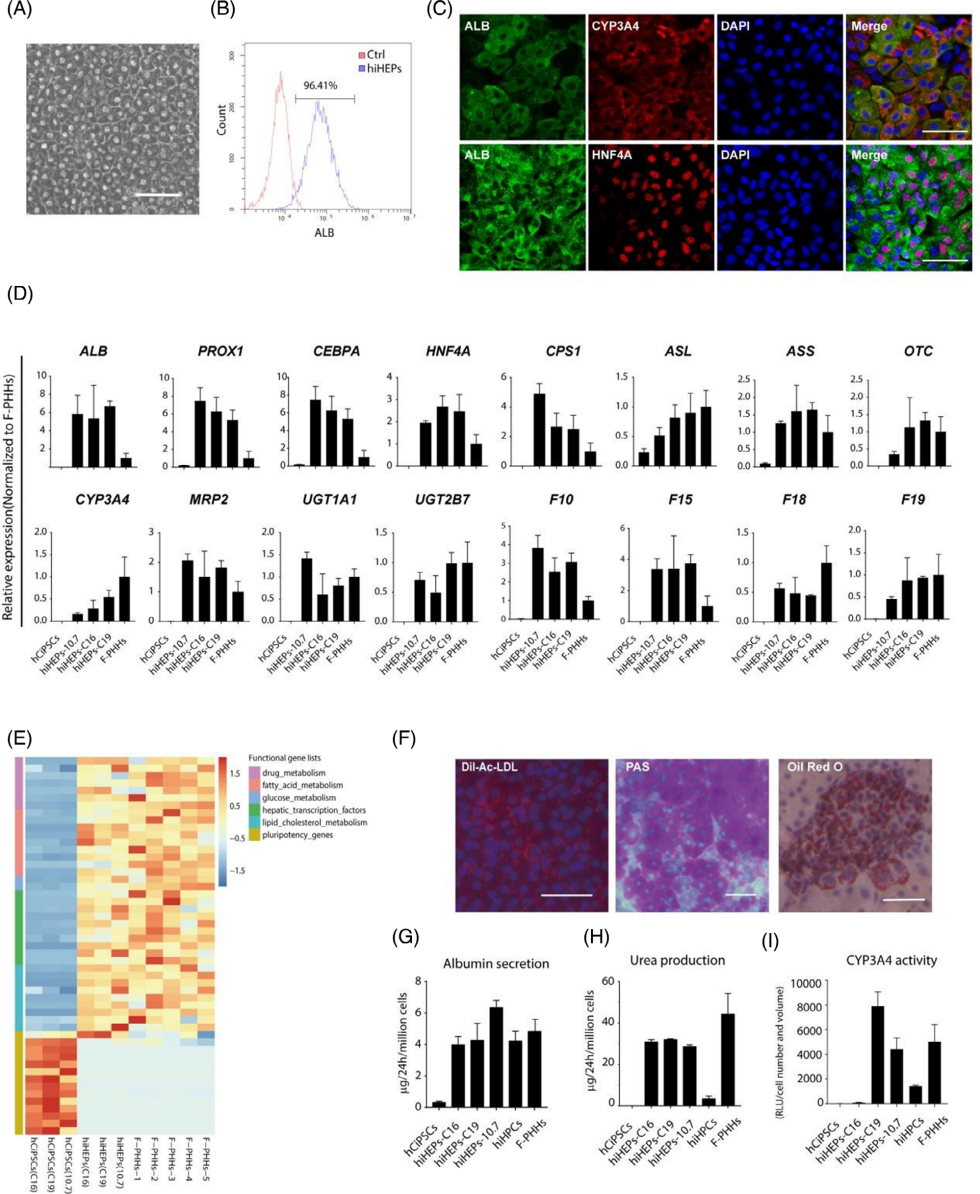
Characterization of hCiPSC‐derived hiHEPs. (A) Brightfield image showing the polygonal morphology of hCiPSC‐derived hiHEPs. Scale bar: 50 μm. (B) Flow cytometry analysis of ALB‐positive hCiPSC‐derived hiHEPs. (C) Co‐immunofluorescence staining of CYP3A4 and HNF4A with ALB in hCiPSC‐derived hiHEPs. Scale bars: 50 μm. (D) RT‐qPCR analysis of major hepatocyte genes in hCiPSCs (*n* = 3), hiHEPs (*n* = 3) derived from different hCiPSC lines, and F‐PHHs (*n* = 3). Relative expression was normalized to PHHs. (E) Heatmap of the expression patterns of functional hepatic lineage‐related genes in hCiPSCs, hiHEPs, and F‐PHHs. (F) Characterization of essential hepatic functions in hiHEPs: LDL uptake (left), PAS staining (middle), and Oil Red O staining (right). Scale bars: 50 μm. (G) ALB secretion in hCiPSCs, hiHEPs, hiHPCs, and F‐PHHs as analysed by ELISA (*n* = 3). (H) Comparison of urea production among hCiPSCs, hiHEPs, hiHPCs, and F‐PHHs (*n* = 3). (I) Comparison of metabolic activity of CYP3A4 among hCiPSCs, hiHEPs, hiHPCs, and F‐PHHs (*n* = 3).

To investigate whether hCiPSC‐derived hiHPCs can be further differentiated into hiHEPs possessing mature functions, the differentiation of hiHPCs was induced using hepatocyte maturation medium for 9 days. hiHPCs differentiated into hepatocytes with polygonal morphology typical of F‐PHHs (Figure 5A). Flow cytometry analysis showed that more than 96% of the differentiated cells expressed the representative hepatocyte marker ALB (Figure 5B). In addition, these differentiated cells also co‐expressed ALB with the key hepatocyte transcription factor HNF4A and phase I cytochrome P450 enzyme CYP3A4 (Figure 5C). Moreover, RT‐qPCR confirmed the expression of multiple marker genes of mature hepatocytes in these cells, including key hepatocyte transcription factors and genes regulating the urea cycle, drug metabolism, and coagulation, the expression levels of which were comparable with those of F‐PHHs (Figure 5D). Furthermore, the expression profiles of genes regulating key hepatocyte functions, such as the metabolism of drugs, fatty acids, glucose, and lipid cholesterol, were similar between hCiPSC‐derived hiHEPs and F‐PHHs by bulk RNA‐seq (Figure 5E). Since hCiPSC‐derived hiHEPs possessed the molecular features of mature hepatocytes, the hepatic function of these cells was further analysed. hCiPSC‐derived hiHEPs were able to take up low‐density lipoprotein (LDL), accumulate lipid droplets, and synthesize glycogen (Figure 5F). Additionally, albumin secretion and urea production in hCiPSC‐derived hiHEPs were at comparable levels to those of F‐PHHs (Figure 5G,H). The metabolic activity of the phase I cytochrome P450 enzyme CYP3A4 in these cells was also similar to that in F‐PHHs (Figure 5I), which is one of the most important functions of mature hepatocytes.^25^ These data indicate that hCiPSC‐derived hiHEPs are similar to mature human hepatocytes in terms of gene expression and hepatic function.

## 4 DISCUSSION

In the present study, we established a stepwise protocol for the efficient in vitro differentiation of hCiPSCs into hiHPCs and hiHEPs. The two‐stage differentiation enhanced the hepatic signature of hepatoblasts and facilitated their differentiation to hiHPCs. Moreover, hiHPCs can be further differentiated into hepatocytes possessing mature functions. Our results demonstrate the feasibility of efficiently generating hiHPCs and functional hiHEPs from hCiPSCs, which sets a foundation for the clinical use of hepatocytes.

It has previously been reported that incompletely differentiated hepatoblasts possessing a deficient gene network failed to differentiate into fully functional hepatocytes.^26^ In our differentiation protocol, the expression of certain key hepatic transcription factors in hepatoblasts at Stage 3 was relatively low, but the addition of another differentiation stage of hepatoblasts significantly enhanced their hepatic properties (Figure 2E), which led to their efficient generation of hiHPCs and shortened the preparation time of high‐purity hepatocytes. On the other hand, the single‐cell data indicated that a proportion of hepatoblasts at Stage 3 already started to express the gene signature of hepatoblasts at stage 4. It could be reasonable that cells timely express hepatic genes along the developmental program during differentiation in vitro. So, it could be possible that extended culturing hepatoblasts in the medium of Stage 3 may also promote the generation of hepatoblasts. The two‐stage induction of hepatoblast in our study represents one feasible way to facilitate the generation of hepatoblasts induced by hCiPSCs.

One important observation was the stage‐specific transcriptomic similarity between hCiPSC‐derived hepatoblasts and human foetal liver cells. scRNA‐seq revealed that hCiPSC‐derived hepatoblasts at Stage 3 and 4 resembled human foetal liver cells at CS12 or PCW5 and CS15–16 or PCW6, respectively (Figure 3B,C). Previous analysis of liver development in vivo confirmed that the differentiation of hepatoblasts consists of two stages, the first of which occurs at PCW5 and the second at PCW6.^17^ These results are consistent with our two‐stage differentiation of hepatoblasts in vitro and suggest that our differentiation protocol recapitulates the process of human liver development, which provides a useful platform for studying the development of human liver in vitro.

Moreover, we show that hCiPSC‐derived hiHEPs resembled F‐PHHs with respect to gene expression and hepatic function. These data, in combination with our recent report regarding the induction of functional islets from hCiPSCs, demonstrate that hCiPSCs are suitable for the generation of different functional cell types for translational medicine.^6^ Considering that the use of small molecules has multiple advantages in translational studies,^5^, ^27^ the application of hCiPSCs to generate functional cell types has great potential for the development of cell therapeutic strategies in a highly controllable and easy‐to‐standardize manner. Our study provides a foundation for the broad application of hCiPSC‐derived hiHEP therapy for the treatment of liver disease and drug discovery in the future.

## AUTHOR CONTRIBUTIONS

Yun Lv and Jun Jia designed the experiments, Yun Lv, Jun Jia, Lulu Liu collected the data. Ziyan Rao, Yun Lv, Chenyang Wu, Yuanyuan Du performed the analysis of bulk RNA‐seq data and scRNA‐seq data. Bei Liu, Jihang Shi and Guangya Li involved in the acquisition of primary cells. Yinan Liu involved in image processing and layout of figures. Yun Lv, Ziyan Rao, Lulu Liu, Jun Xu, Dongyu Zhao and Hongkui Deng involved in the preparation the manuscript. Hongkui Deng and Dongyu Zhao supervised the research.

## FUNDING INFORMATION

This work was supported by National Natural Science Foundation of China (32288102) to H. Deng; and by National Key Research and Development Program of China 2021YFF1201100 and National Natural Science Foundation of China (32270603) to D. Zhao. Meanwhile, this work was also supported by Changping Laboratory.

## CONFLICT OF INTEREST STATEMENT

All authors confirm that there are no conflicts of interest.

## Supporting information


**Figure S1.** Characterization of hiHPCs and hiHEPs differentiated from iPSCs induced by Yamanaka factors.
**Figure S2.** The cell fate of hepatoblasts is further specialized from Stage 3 to stage 4.
**Figure S3.** UMAP plots of scTourtrajectory inference of the top 20 inferred hepatic driver genes whose expression tended to increase from Stage 3 to stage 4.
**Figure S4.** Liver fate is specialized from CS12 to CS15–16 in vivo.
**Figure S5.** Gene ontology biological process enrichment of each hepatic lineage‐related regulon at Stage 3.
**Figure S6.** Gene ontology biological process enrichment of each hepatic lineage‐related regulon at stage 4.

**Table S1.** Antibody list.
**Table S2.** Primers used for qPCR.

## Data Availability

All data are available in the Article and its Supplementary Information. The next‐generation sequencing datasets have been deposited to the Gene Expression Omnibus (GEO) with the accession GSE233413 (secure token: kxejokikpbwpnof). The public datasets we used can be accessed at NCBI GEO database under accession numbers GSE112330 (F‐PHHs, hFLCs), GSE157329 (CS samples). PCW samples is available athttps://collections.cellatlas.io/liver-development.
