# Supplementary material for: The efficient generation of functional human hepatocytes from chemically induced pluripotent stem cells
Source: Cell Prolif. 2023 Oct 9;57(2):e13540. doi: 10.1111/cpr.13540 (PMC10849784; doi:10.1111/cpr.13540)
Supplement: Supplementary file 1 — Figure S1. Characterization of hiHPCs and hiHEPs differentiated from iPSCs induced by Yamanaka factors. Figure S2. The cell fate of hepatoblasts is further specialized from Stage 3 to stage 4. Figure S3. UMAP plots of scTourtrajectory inference of the top 20 inferred hepatic driver genes whose expression tended to increase from Stage 3 to stage 4. Figure S4. Liver fate is specialized from CS12 to CS15–16 in vivo. Figure S5. Gene ontology biological process enrichment of each hepatic lineage‐related regulon at Stage 3. Figure S6. Gene ontology biological process enrichment of each hepatic lineage‐related regulon at stage 4. [file CPR-57-e13540-s002.docx]

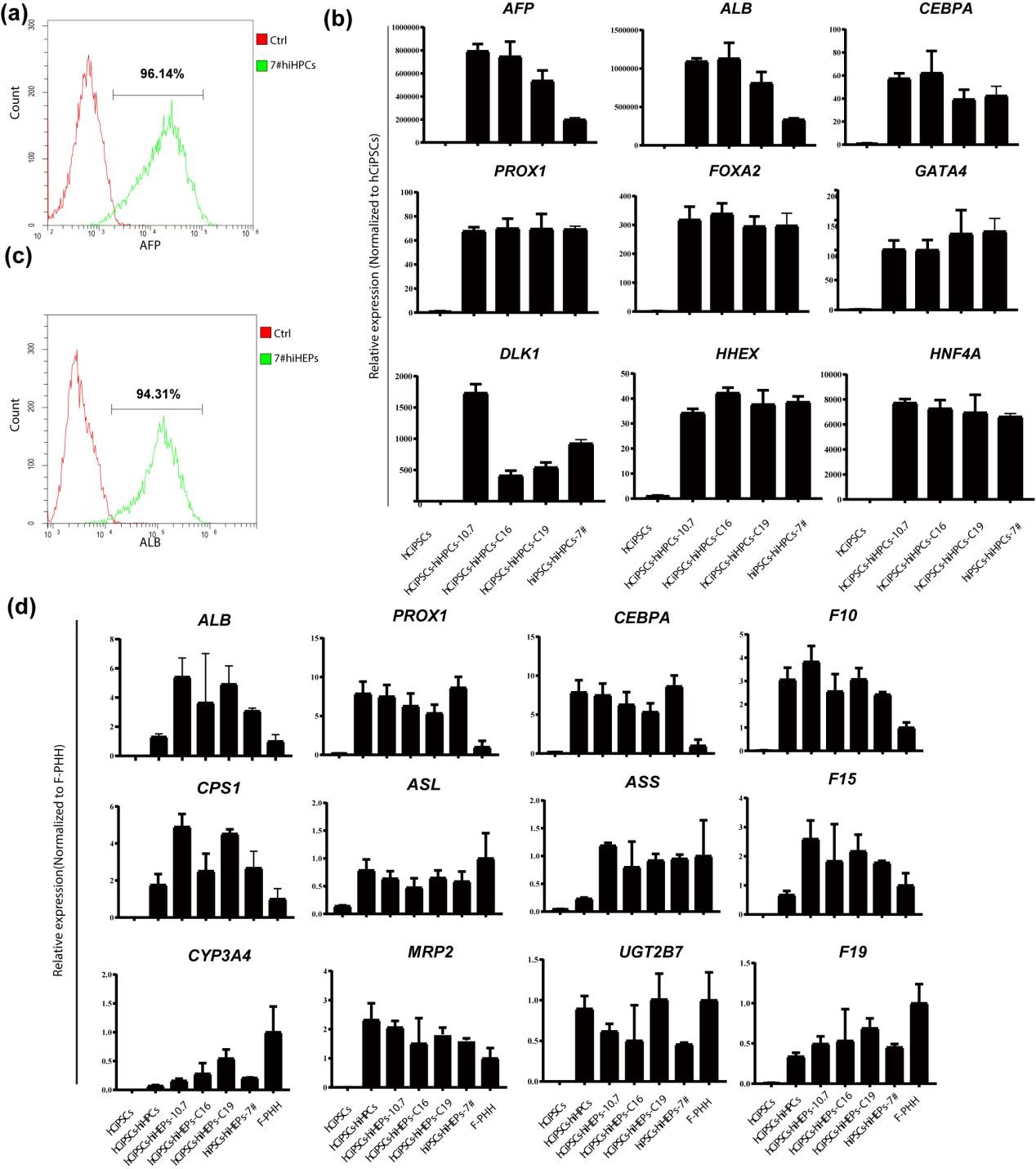


**Figure S1. Characterization of hiHPCs and hiHEPs differentiated from iPSCs induced by Yamanaka factors.**

1. Flow cytometry analysis of AFP-positive hiPSCs-7#-derived hiHPCs.
2. RT-qPCR analysis of the major hepatic progenitor genes in hCiPSCs (n = 3), hiHPCs (n = 3) derived from different hCiPSC lines, hiPSCs-7#-derived hiHPCs (n = 3). Relative expression was normalized to hCiPSCs.
3. Flow cytometry analysis of ALB-positive hiPSCs-7#-derived hiHEPs.
4. RT-qPCR analysis of major hepatocyte genes in hCiPSCs (n = 3), hCiPSCs-derived hiHPCs (n = 3), hiHEPs (n = 3) derived from different hCiPSC lines, hiPSCs-7#-derived hiHEPs (n = 3) and F-PHHs (n = 3). Relative expression was normalized to F-PHHs.


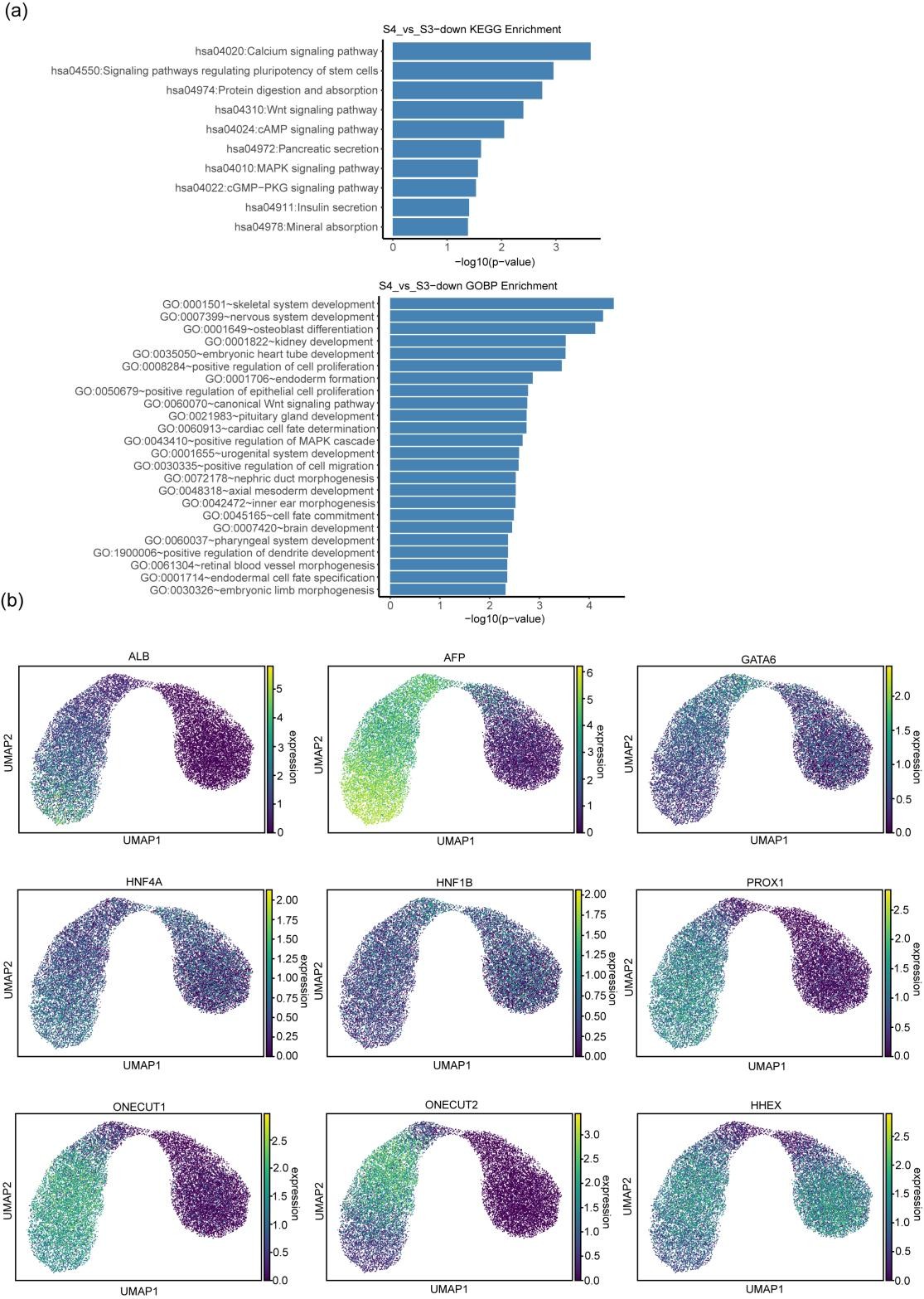


**Figure S2. The cell fate of hepatoblasts is further specialized from stage 3 to stage 4.**

1. KEGG (up) and gene ontology biological process (down) enrichment bar graph of the 516 downregulated genes at S4 as compared with S3. The x-axis represents the -log10 p-value.
2. UMAP plots of hepatic marker expression. The expression of each gene was log-normalized.


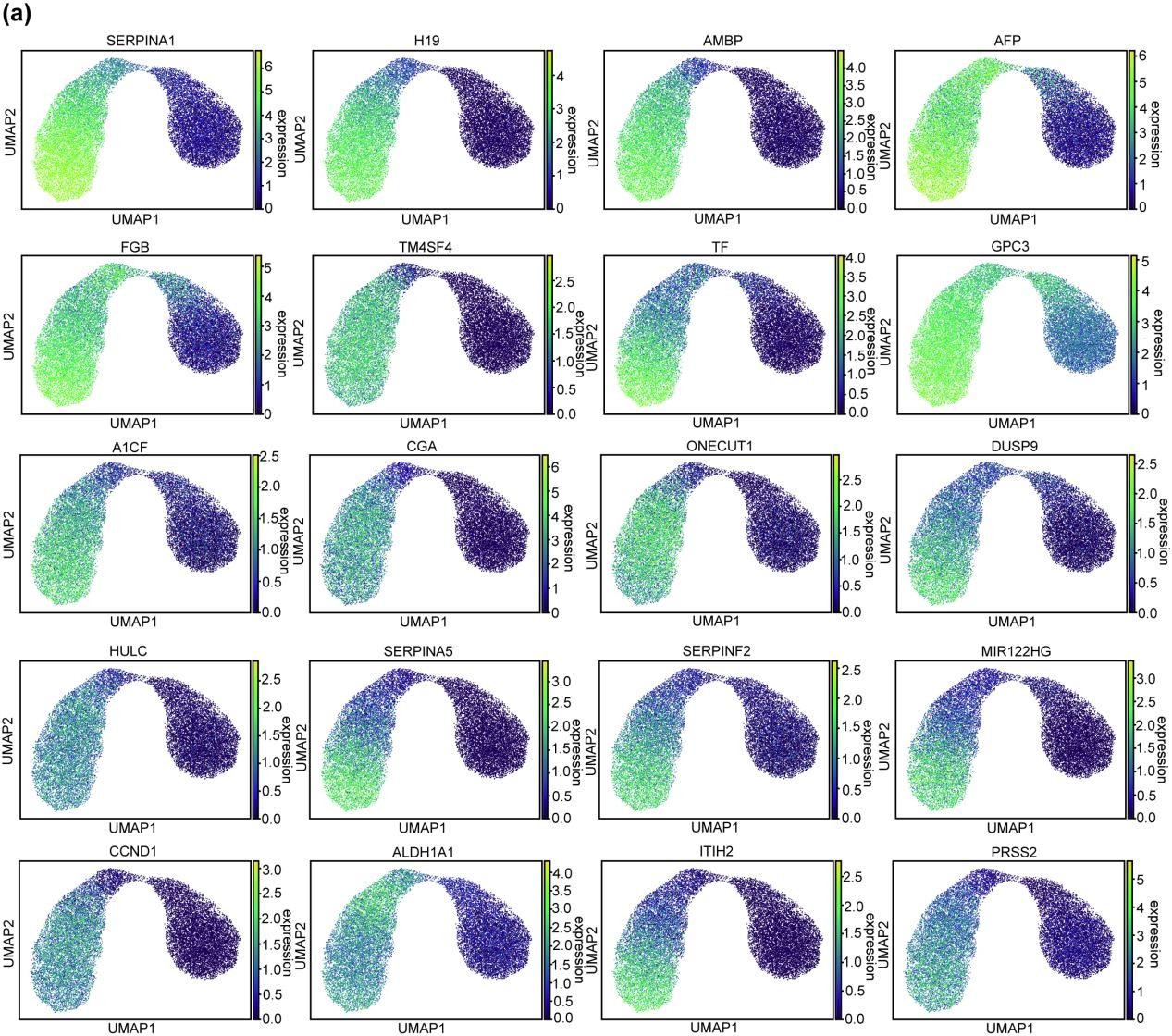


**Figure S3. UMAP plots of scTour trajectory inference of the top 20 inferred hepatic driver genes whose expression tended to increase from stage 3 to stage 4.**


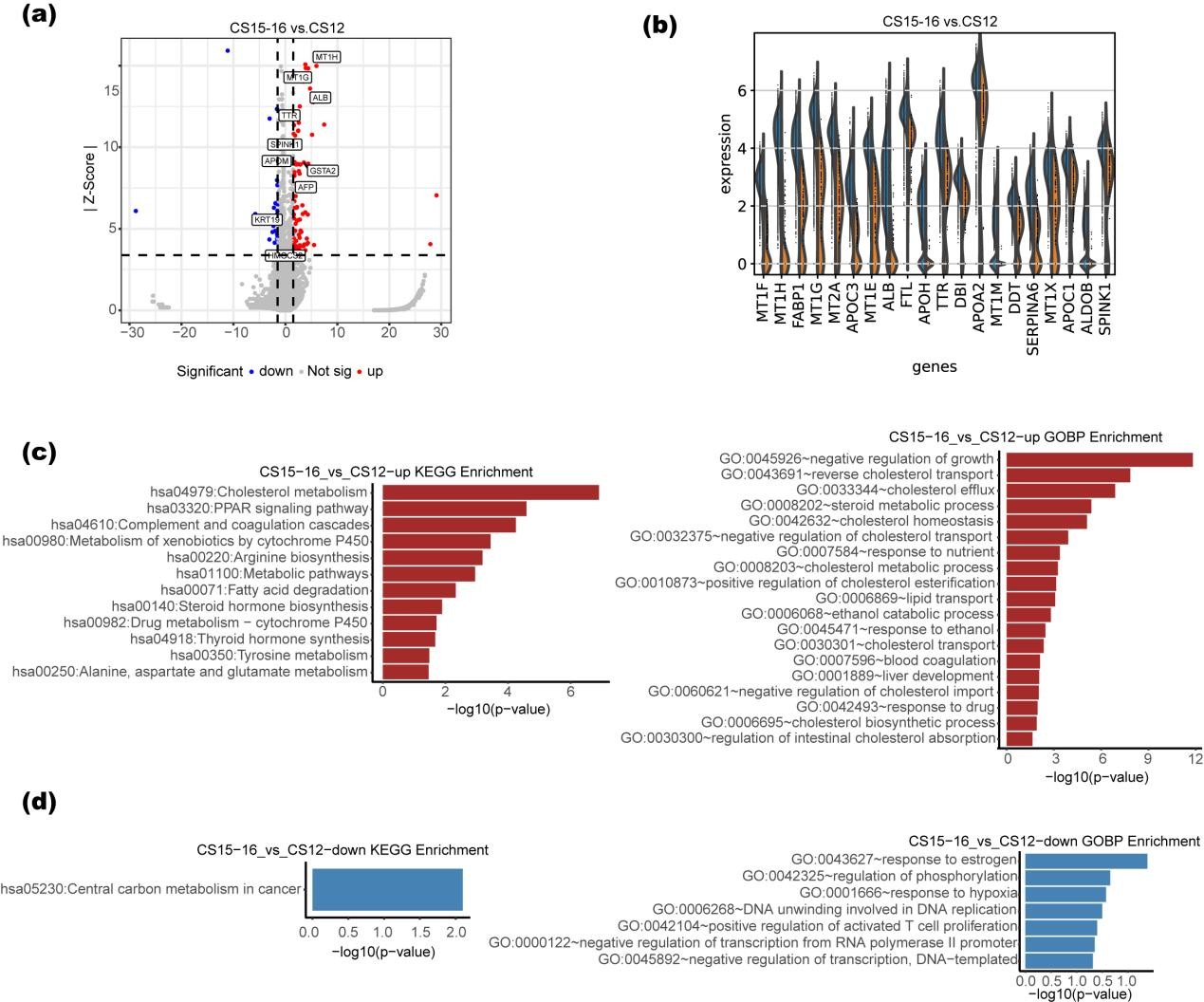


**Figure S4. Liver fate is specialized from CS12 to CS15–16 *in vivo*.**

1. Volcano plot of the overall differentially expressed genes between CS12 and CS15–16. Red dots represent the 92 upregulated genes, while blue dots represent the 30 downregulated genes at CS15–16 as compared with CS12. The identified genes were hepatic lineage-related.

|logFC| > 1.5, wilcoxon rank-sum test adjusted p-value < 0.05.

1. Violin plot of the top 20 upregulated genes at CS15–16 as compared with CS12, ranked by z-score.
2. KEGG (left) and gene ontology biological process (right) enrichment bar graph of the 92 upregulated genes at CS15–16 as compared with CS12. The x-axis represents the -log10 p-value.
3. KEGG (left) and gene ontology biological process (right) enrichment bar graph of the 30 downregulated genes at CS15–16 as compared with CS12. The x-axis represents the -log10 p-value.


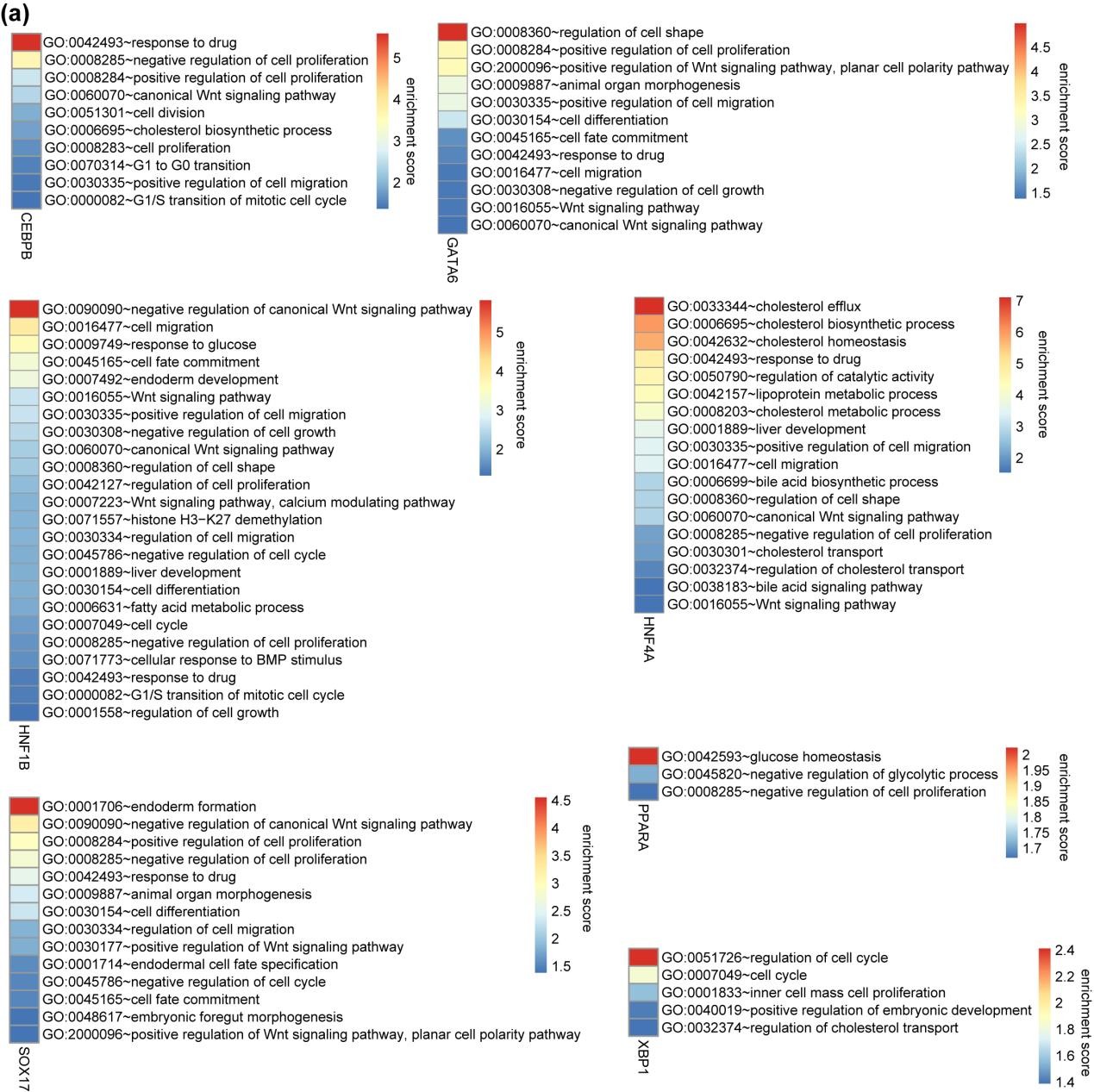


**Figure S5. Gene ontology biological process enrichment of each hepatic lineage-related regulon at stage 3.**


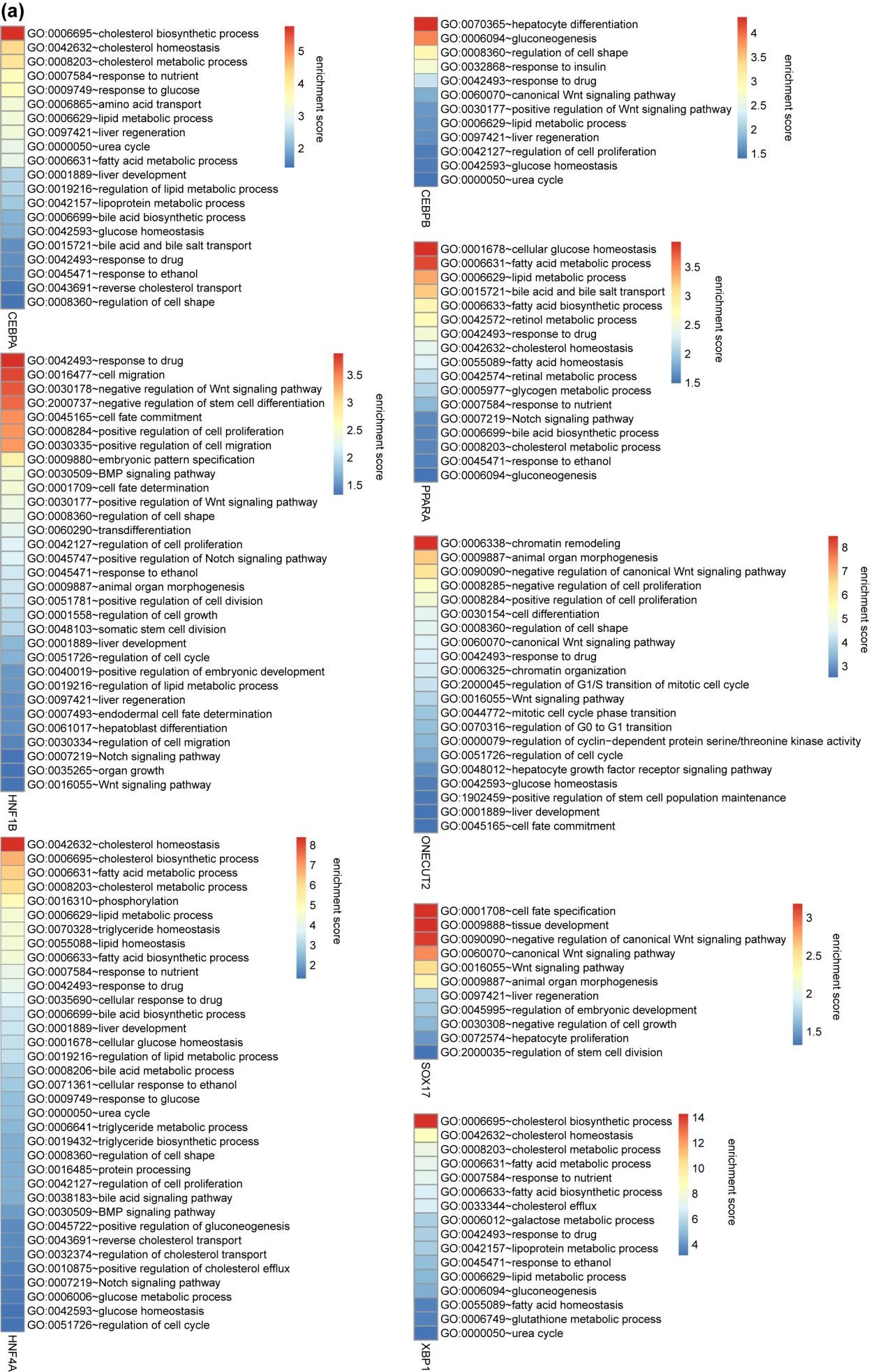


**Figure S6. Gene ontology biological process enrichment of each hepatic lineage-related regulon at stage 4.**
