# Supplementary material for: The efficient generation of functional human hepatocytes from chemically induced pluripotent stem cells
Source: Cell Prolif. 2023 Oct 9;57(2):e13540. doi: 10.1111/cpr.13540 (PMC10849784; doi:10.1111/cpr.13540)
Supplement: Supplementary file 2 — Table S1. Antibody list. Table S2. Primers used for qPCR. [file CPR-57-e13540-s001.docx]

**Supplemental Table 1. Antibody list**

| Antibody | Supplier | Catalog No. |
| --- | --- | --- |
| AFP | invitrogen | MA5-14666 |
| AFP | DAKO | A0008 |
| ALB | Bethy | A80-129A |
| HNF4A | cell signaling | 3113s |
| CYP3A4 | invitrogen | MA5-17064 |
| CK18 | ORIGENE | TA500015 |
| KI67 | abcam | ab243878 |
| rb488 | abcam | ab96891 |
| rb550 | abcam | ab98489 |
| rb647 | abcam | ab96922 |
| ms488 | abcam | ab96875 |
| ms647 | abcam | ab98797 |
| gt550 | abcam | ab96932 |
| gt647 | abcam | ab96938 |

**Supplemental Table 2. Primers used for qPCR**

| Gene | Forward Primer (5'-3') | Reverse Primer (5'-3') |
| --- | --- | --- |
| AFP | CCCGAACTTTCCAAGCCATA | TACATGGGCCACATCCAGG |
| ALB | GCACAGAATCCTTGGTGAACAG | ATGGAAGGTGAATGTTTCAGCA |
| CEBPA | ACAAGAACAGCAACGAGTACCG | CATTGTCACTGGTCAGCTCCA |
| DLK1 | GGGCACAGGAGCATTCATAG | GACGGGGAGCTCTGTGATAG |
| FOXA2 | CGACTGGAGCAGCTACTATGC | TACGTGTTCATGCCGTTCAT |
| GATA4 | CCCGACACCCCAATCTC | CAGGCGTTGCACAGATAGTG |
| HHEX | ACGGTGAACGACTACACGC | CGTTGGAGAATCTCACCTGG |
| HNF4A | ACTACATCAACGACCGCCAGT | ATCTGCTCGATCATCTGCCAG |
| PROX1 | ACAGGGCTCTGAACATGCAC | GGCATTGAAAAACTCCCGTA |
| CPS1 | AATGAGGTGGGCTTAAAGCAAG | AGTTCCACTCCACAGTTCAGA |
| ASL | CAGTGGACCCCATCATGGAGA | GGCTTTGCTGCCTTGAACATC |
| OTC | CGGCCCGTGTATTGTCTAGC | TAGCCAGGGTGTCCAAATCTG |
| ASS | CTTGGGGCCAAAAAGGTGTTC | GAGGTAGCGGTCCTCATACAG |
| F10 | CACTGGTCGCCATCTTTGTA | AGTGCATGGAAGAGACCTGC |
| F15 | CATGGCACCTGCACAAGATG | ATGGCCCCAGTCATGTCTAC |
| F18 | CAACGCCAAGTTCGCTTTCA | AATGCCAACGGGTGCTATGA |
| F19 | GAGAAGAAGCGCAGTCACCT | TCTTCCCATCAATGAGCCGC |
| CYP3A4 | GGTGGTGAATGAAACGCTCAG | ACCCCTTTGGGAATGAACATC |
| MRP2 | GGGATCTCTTCCACACTGGAT | CATACAGGCCCTGAAGAGGA |
| UGT1A1 | CCATCATGCCCAATATGGTT | CCACAATTCCATGTTCTCCA |
| UGT2B7 | AACGTAATTGCATCAGCCCT | GGTCATTCTGGGGTATCCAC |
